# Screening for harmful substance use in emergency departments: a systematic review

**DOI:** 10.1186/s12245-024-00616-2

**Published:** 2024-04-08

**Authors:** Jessica Moe, Justin Koh, Jennifer A. Ma, Lulu X. Pei, Eleanor MacLean, James Keech, Kaitlyn Maguire, Claire Ronsley, Mary M. Doyle-Waters, Jeffrey R. Brubacher

**Affiliations:** 1 Department of Emergency Medicine, University of British Columbia, Diamond Health Care Centre, 11 Floor – 2775 Laurel Street, Vancouver, BC V5Z 1M9 Canada; 2 Department of Emergency Medicine, Queen’s University, Kingston Health Sciences Centre, 76 Stuart Street, Kingston, ON K7L 2V7 Canada; 3 Department of Emergency Medicine, University of Manitoba, S203 Medical Sciences Building, 750 Bannatyne Avenue, Winnipeg, MB R3E 0W2 Canada; 4 School of Medicine, Queen’s University, 15 Arch Street, Kingston, ON K7L 3N6 Canada; 5 Centre for Clinical Epidemiology and Evaluation, Vancouver Coastal Health Research Institute, 7th Floor, 828 West 10th Avenue, Research Pavilion, Vancouver, BC V5Z 1M9 Canada

**Keywords:** Substance-related disorders, Drug abuse screening, Emergency, Departments, Public health

## Abstract

**Background:**

Substance use-related emergency department (ED) visits have increased substantially in North America. Screening for substance use in EDs is recommended; best approaches are unclear. This systematic review synthesizes evidence on diagnostic accuracy of ED screening tools to detect harmful substance use.

**Methods:**

We included derivation or validation studies, with or without comparator, that included adult (≥ 18 years) ED patients and evaluated screening tools to identify general or specific substance use disorders or harmful use. Our search strategy combined concepts *Emergency Department* AND *Screening* AND *Substance Use*. Trained reviewers assessed title/abstracts and full-text articles for inclusion, extracted data, and assessed risk of bias (QUADAS-2) independently and in duplicate. Reviewers resolved disagreements by discussion. Primary investigators adjudicated if necessary. Heterogeneity precluded meta-analysis. We descriptively summarized results.

**Results:**

Our search strategy yielded 2696 studies; we included 33. Twenty-one (64%) evaluated a North American population. Fourteen (42%) applied screening among general ED patients. Screening tools were administered by research staff (*n* = 21), self-administered by patients (*n* = 10), or non-research healthcare providers (*n* = 1). Most studies evaluated alcohol use screens (*n* = 26), most commonly the Alcohol Use Disorders Identification Test (AUDIT; *n* = 14), Cut down/Annoyed/Guilty/Eye-opener (CAGE; *n* = 13), and Rapid Alcohol Problems Screen (RAPS/RAPS4/RAPS4-QF; *n* = 12). Four studies assessing six tools and screening thresholds for alcohol abuse/dependence in North American patients (AUDIT ≥ 8; CAGE ≥ 2; Diagnostic and Statistical Manual of Mental Disorders, 4th Edition [DSM-IV-2] ≥ 1; RAPS ≥ 1; National Institute on Alcohol Abuse and Alcoholism [NIAAA]; Tolerance/Worry/Eye-opener/Amnesia/K-Cut down [TWEAK] ≥ 3) reported both sensitivities and specificities ≥ 83%. Two studies evaluating a single alcohol screening question (SASQ) (*When was the last time you had more than X drinks in 1 day?, X* = *4 for women; X* = *5 for men)* reported sensitivities 82–85% and specificities 70–77%. Five evaluated screening tools for general substance abuse/dependence (Relax/Alone/Friends/Family/Trouble [RAFFT] ≥ 3, Drug Abuse Screening Test [DAST] ≥ 4, single drug screening question, Alcohol, Smoking and Substance Involvement Screening Test [ASSIST] ≥ 42/18), reporting sensitivities 64%-90% and specificities 61%-100%. Studies’ risk of bias were mostly high or uncertain.

**Conclusions:**

Six screening tools demonstrated both sensitivities and specificities ≥ 83% for detecting alcohol abuse/dependence in EDs. Tools with the highest sensitivities (AUDIT ≥ 8; RAPS ≥ 1) and that prioritize simplicity and efficiency (SASQ) should be prioritized.

**Supplementary Information:**

The online version contains supplementary material available at 10.1186/s12245-024-00616-2.

## Background

Emergency departments (EDs) provide a crucial opportunity to screen for substance use disorders and to provide essential care for people with substance use-related concerns [1]. In the last decade, ED visits related to substance use have increased substantially in North America [2–4]. Annual ED and inpatient costs of substance use disorder exceeded $13 billion in 2017 in the United States, and in Canada, per capita costs increased from $321 to $353 between 2007 and 2020 [5, 6]. Between 2014 and 2018, there were 9.3 million ED visits by patients with substance use disorders in the United States; during this period, substance-use related visits increased by 30% relative to baseline. Overall, one in 11 patients visiting an ED had a co-morbid alcohol or other substance use disorder [3]. Alcohol-related ED visits increased by 47% between 2006 and 2014 in the United States [4]. Similarly, in the Canadian province of Ontario, ED visits for alcohol-related concerns increased by 440% from 2003 to 2016 [7]. In the Canadian province of Alberta, ED visits related to any substance use increased by 38%, and those related to opioid use increased by 57.3% from 2010 to 2015 [8]. Alberta also saw a 168% increase in ED visits related to stimulant use from 2010 to 2017 [9]. These statistics emphasize the enormous and increasing impact of substance use in EDs, and the important role that emergency physicians play in identification, risk stratification, and management of substance use disorders.

ED visits are key contact points with high-risk patients with substance use and opportunities to provide life-saving interventions [1]. Data from British Columbia, Canada indicate that 60% of people who overdosed in 2015–2016 visited an ED in the year prior to their overdose event [10]. This highlights that ED visits are critical, and often missed, opportunities to identify at-risk individuals. The ED provides a unique opportunity for screening and identification of harmful substance use, initiating pharmacological and psychosocial interventions, and making referrals to outpatient addictions care [11]. A 2017 American College of Emergency Physicians position statement affirmed that “emergency medical professionals are positioned and qualified to mitigate the consequences of alcohol abuse through screening programs, brief intervention, and referral to treatment” [12]. This was similarly reflected in a 2020 position statement from the Canadian Association of Emergency Physicians, which recommended that emergency providers use case-finding strategies to identify opioid and other substance use disorders [13].

Although the need and opportunity for ED substance use screening is recognized and endorsed, there is no accepted recommendation on preferred screening methods. Numerous screening tools have been adapted for use in EDs, however their comparative performance is poorly understood. Determining the most sensitive and specific screening tools to identify individuals with harmful substance use will provide crucial information to inform ED guidelines and recommendations for best practices.

## Methods

### Aim

The primary objective of this systematic review is to synthesize available research to identify the diagnostic accuracy of screening tools in detecting harmful substance use and substance use disorders in an ED setting. We evaluated screening tools designed to detect both general and specific substance use-related harms.

### Design

Our systematic review meets 2020 Preferred Reporting Items for Systematic Reviews and Meta-Analyses (PRISMA) guidelines [14].

### Registration and protocol

We submitted a systematic review protocol to PROSPERO prior to study initiation. Due to COVID-19-related delays, our protocol was not published prior to completion of our review.

### Eligibility criteria

We included studies evaluating the following:

- *Population:* Adults (≥ 18 years) presenting to EDs in any country, for any reason.
- *Intervention:* Screening tools to identify substance use disorders or harmful use, for general or specific substances.
- *Outcomes:* Identification or diagnosis of a substance use disorder or harmful substance use-related health outcome.
- *Study Design:* derivation and/or validation studies with or without a comparator group. We only included interventional studies with integrated screening if they evaluated accuracy of screening compared to a gold standard substance use-related diagnosis. We excluded reviews.

### Information sources and search strategy

We developed a search strategy that combined concepts *Emergency Department* AND *Screening* AND *Substance Use* using Medical Subject Headings (MeSH), keywords, and author-assigned terms informed by a previous literature review [11]. Studies referenced in this review were hand-searched for potential eligibility in the present systematic review. We limited our search to publications on or after January 1, 2000, adults, English language, and human studies. We applied our search to Medical Literature Analysis and Retrieval System Online (MEDLINE [Ovid]) and Embase (Ovid) (to January 5, 2021), PsycINFO (EBSCO) (to February 8, 2021), HaPI – Health and Psychosocial Instruments database (Ovid) (to February 8, 2021), Web of Science (Clarivate Analytics) (to February 26, 2021), and CINAHL – Cumulative Index to Nursing and Allied Health Literature (EBSCO) (to March 10, 2021). We report our full MEDLINE search strategy in Additional file 1.

### Selection and data collection

We exported citations into Covidence and removed duplicates [15]. Two trained reviewers independently assessed title/abstracts (CR, KM) and excluded articles that were obviously irrelevant. Each potentially eligible title/abstract underwent full-text eligibility review by two of five authors independently and in duplicate (JMa, EM, JKe, KM, CR). For both title/abstract and full-text screening, reviewers met to discuss eligibility decisions after assessing an initial 20 citations to ensure consistency, then completed review of the remaining citations independently. Reviewers resolved disagreements by discussion, with primary investigators (JMo, JKo) adjudicating if they could not reach consensus.

Two of four reviewers then extracted data independently and in duplicate from eligible articles (JMa, EM, JKe, JMo). Reviewers completed all assessments and extractions using standardized forms that were pilot tested among independent research colleagues for face validity. Reviewers discussed and resolved discrepancies and involved the primary investigators (JMo, JKo) to adjudicate when they could not reach consensus. When data were missing or ambiguous, we emailed authors up to two times to request additional information.

### Data items

We extracted information relating to the study characteristics (authors, publication year, design, location, time period, follow-up period if applicable, data sources); study participants (inclusion/exclusion criteria, age, sex, gender, ethnicity, education, occupational status, marital status, income, ED presentation, comorbidities, number of participants in main analysis, losses to follow-up); details about screening (methods of determining eligibility, screening tools, substances screened for, person administering screening, person interpreting screening results, follow-up after ED visit, and descriptions of associated interventions where applicable); and patient outcomes (definitions/thresholds for “screen positive,” numbers screened positive and negative, gold standard definition, methods of ascertainment, person applying the screening tool and gold standard, sensitivity and specificity of the screening tool).

### Study risk of bias

We assessed risk of bias in included studies using the Quality Assessment of Diagnostic Accuracy Studies-2 (QUADAS-2) tool specifically developed for diagnostic accuracy studies [16]. QUADAS-2 comprises four domains of patient selection, index test, reference standard, and flow and timing. Two of four reviewers (JMa, EM, JKe, JMo) performed assessments independently and in duplicate. Reviewers met to discuss risk of bias decisions after an initial 12 appraisals to ensure consistency, then completed the assessments independently. Reviewers resolved discrepancies by discussion and involved the primary investigators (JMo, JKo) to adjudicate if they could not reach consensus.

### Effect measures

For the diagnosis of a substance use disorder or a substance use-related patient outcome, effect measures were diagnostic accuracy (e.g., sensitivity, specificity).

### Synthesis methods

We attempted to group papers assessing identical screening tools, “screen positive” thresholds, substances, and gold standard outcome definitions to meta-analyze sensitivity and specificity in ED populations. Due to a limited number of studies that could be grouped, we were unable to proceed with meta-analysis.

We descriptively summarized results in forest plots. We limited our visual summaries to studies assessing outcomes of alcohol abuse and/or dependence since this comprised the majority, and to North American studies to support comparability across screening tools. We did not limit our visual summaries to studies with low risk of bias, as many performed variably on the distinct domains of the QUADAS-2 tool. We instead present all studies meeting the above criteria, along with risk of bias assessments (Table 2), enabling clinicians and decision makers to interpret summative visual results in the context of studies’ quality assessments. We extracted information from each study to generate a 2 × 2 table and used the R package “meta” to obtain 95% confidence intervals for sensitivity and specificity. We used the Clopper-Pearson method to calculate confidence intervals. We plotted these values for outcomes of Diagnostic and Statistical Manual of Mental Disorders, 4th Edition (DSM-IV) alcohol abuse and/or dependence, as these were the reference standards most commonly reported in the included studies meeting criteria for visual summarization.

## Results

### Study selection

**Fig. 1:**
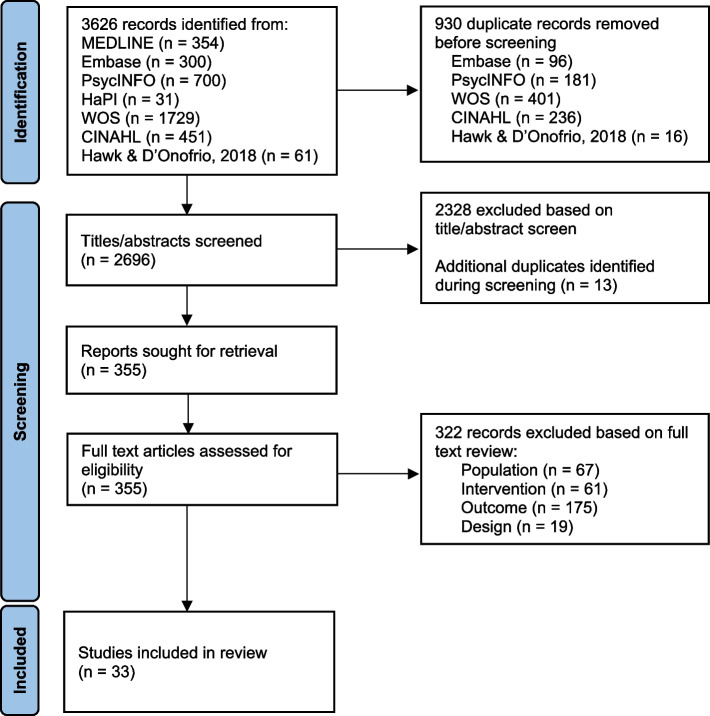
Study flow diagram

After removing duplicates, our search strategy yielded 2696 citations. We excluded 2328 after title/abstract review and 322 after full-text review, most commonly for ineligible outcome (*n* = 175) and population (*n* = 67). Five of 33 included articles evaluated subsets of the same population. For these studies, we included data evaluating the same tool(s) only once [17–21]. See Fig. 1 for the study flow diagram.

### Study characteristics

We summarize study characteristics, including participants, screening tools, “screen positive” cut-offs, reference standard, and tool performance in Table 1. We report on 33 included studies [17–49]. All were cohort studies. Twenty-one studies (64%) evaluated a North American population. A minority of studies were set in Europe (*n* = 6), South America (*n* = 4 [N.B. some overlap with North American studies]), Asia (*n* = 2), and Africa [1]. Studies included a median of 530 patients (IQR: 200, 1492). Fourteen studies (42%) applied screening among a general ED population, whereas others evaluated patients with specific presentations (trauma/injury [*n* = 6], psychiatric [*n* = 3], alcohol intoxication [*n* = 2], opioid prescription request and/or pain [*n* = 3]). Three studies applied further screening to ED patients who reported alcohol use in the last 12 months [30, 31, 45]. Where reported, tools were applied by patient surveys or questionnaires (*n* = 10), or by interviewers with research or clinical backgrounds (*n* = 22). In most studies, trained research staff conducted screening (*n* = 21); in one study, physicians on duty administered the screening tool.

**Table 1:** Study characteristics and screening tool performance

Author, Year	Location	Study design	Participants (n)	Screening tool (cut-off)	Method of administration	Substance(s) addressed	Reference standard	Sensitivity	Specificity
Bastiaens, 2002 [22]	USA	Prospective cohort	Adult patients presenting to a psychiatric emergency and evaluation centre between 8am-8 pm during study period (*n* = 215)	RAFFT (3)	Surveys administered by psychiatric triage nurses	Multiple drugs	DSM-IV substance use disorder (MINI)	0.84	0.67
				RAFFT (3)		Alcohol	DSM-IV alcohol abuse/dependence (MINI)	0.73	0.71
				CAGE (2)				0.90	0.84
Beaudoin, 2016 [23]	USA	Prospective cohort	ED patients ≥ 50 years who have used an opioid prescription within the past 30 days (*n* = 113)	PDUQp (10)	Self-report on electronic tablet computer	Opioids	Opioid misuse (NESARC)	0.44	0.79
				PDUQp (10)			DSM-V opioid use disorder (AUDADIS)	0.38	0.81
				PDUQp (10)			DSM-V opioid use disorder (moderate to severe) (AUDADIS)	0.56	0.75
Borges, 2001 [24]	Santa Clara, San Jose (USA) and Pachuca, Mexico	Prospective cohort	Hispanic ED patients ≥ 18 years (*n* = 586 [Santa Clara]; 1511 [Pachuca])	RAPS (1)	Trained interviewers	Alcohol	ICD-10 and DSM-IV alcohol dependence (CIDI)	0.93	0.79
				Optimized screening tool generated from other tools (CAGE, BMAST, AUDIT, TWEAK, TRAUMA)				0.98	0.65
				RAPS (1)			ICD-10 and DSM-IV alcohol dependence and harmful drinking/abuse (CIDI)	0.74	0.85
				Optimized screening tool generated from other tools (CAGE, BMAST, AUDIT, TWEAK, TRAUMA)				0.89	0.71
Brousse, 2014 [25]	France	Prospective cohort	ED patients 18–80 years presenting with acute alcohol intoxication as principal or additional diagnosis AND blood alcohol level ≥ 0.8 g/L (*n* = 164)	AUDIT (12 [Male])	Trained interviewers	Alcohol	DSM-IV alcohol abuse (MINI)	PPV: 1.00^a^	NPV: 0.42
				AUDIT (7 [Female])				PPV: 0.95	NPV: 1.00
				RAPS4-QF (2 [Male])				PPV: 0.97	NPV: 1.00
				CAGE (2 [Female])				PPV: 0.95	NPV: 1.00
				AUDIT (14 [Male])			DSM-IV mild alcohol dependence (MINI)	PPV: 0.93	NPV: 0.55
				AUDIT (11 [Female])				PPV: 0.86	NPV: 1.00
				CAGE (3 [Female])				PPV: 0.88	NPV: 0.80
				RAPS4-QF (3 [Male])				PPV: 0.90	NPV: 0.60
				AUDIT (18 [Male])			DSM-IV moderate alcohol dependence (MINI)	PPV: 0.94	NPV: 0.50
				AUDIT (18 [Female])				PPV: 0.96	NPV: 0.65
Canagasaby, 2005 [26]	USA	Prospective cohort	ED patients ≥ 18 years presenting within 48 h of acute injury (*n* = 2517 cases); ED patients ≥ 18 years presenting for care of medical illness, matched by age group, sex, and rural versus urban (*n* = 1151 medical controls)	SASQ (5 drinks in last 3 months [men])	Trained interviewers	Alcohol	DSM-IV current alcohol use and dependence (DIS)	0.85	0.70
				SASQ (4 drinks in last 3 months [women])				0.82	0.77
				Quantity-Frequency (8 [men])				0.78	0.78
				Quantity-Frequency (6 [women])				0.78	0.78
				Quantity (3 [men])				0.81	0.66
				Quantity (3 [women])				0.71	0.76
Chalmers, 2019 [27]	USA	Prospective cohort	ED patients ≥ 18 years requesting an opioid prescription refill, with chronic pain ≥ 6 months, or experiencing pain at time of presentation (*n* = 719)	COMM (9)	Research assistants	Opioids	Any of 10 aberrant behaviours noted in EMR	0.49	0.51
				ORT (8)				0.93	0.60
				SOAPP-R (18)				0.57	0.45
				COMM (9)			Any of 10 aberrant behaviours noted in EMR OR statewide prescription drug monitoring database OR local medical examiner database	0.50	0.51
				ORT (8)				0.38	0.59
				SOAPP-R (18)				0.60	0.50
Cherpitel, 2000a [19]	Pachuca, Mexico and Santa Clara, USA	Prospective cohort	Hispanic ED patients ≥ 18 years (*n* = 1511 [Pachuca, 93% of 1624 patients sampled]; 586 [Santa Clara, 80% of 733 Hispanic patients sampled])	CAGE (2 [Pachuca])	Interviews by trained research assistants	Alcohol	ICD-10 current harmful drinking or alcohol dependence, and DSM-IV current alcohol abuse or dependence (CIDI)	0.76	0.94
				CAGE (2 [Santa Clara])				0.88	0.90
				BMAST (6 [Pachuca])				0.42	0.99
				BMAST (6 [Santa Clara])				0.71	0.97
				AUDIT (8 [Pachuca])				0.92	0.93
				AUDIT (8 [Santa Clara])				0.95	0.90
				TWEAK (8 [Pachuca])				0.90	0.91
				TWEAK (3 [Santa Clara])				0.91	0.88
				RAPS (1 [Pachuca])				0.92	0.93
				RAPS (1 [Santa Clara])				0.95	0.88
				Trauma Scale (2 [Pachuca])				0.31	0.95
				Trauma Scale (2 [Santa Clara])				0.67	0.81
				Breathalyzer (positive [Pachuca])				0.51	0.93
				Breathalyzer (positive [Santa Clara])				0.21	0.99
				REPORT drinking within 6 h prior to event (yes [Pachuca])				0.45	0.93
				REPORT drinking within 6 h prior to event (yes [Santa Clara])				0.45	0.95
				5 MONTHLY (yes [Pachuca])				0.85	0.91
				5 MONTHLY (yes [Santa Clara])				0.81	0.88
				HOLD (5 [Pachuca])				0.96	0.76
				HOLD (5 [Santa Clara])				0.89	0.74
				Arrests for driving after drinking (yes [Pachuca])				0.07	1.00
				Arrests for driving after drinking (yes [Santa Clara])				0.50	0.88
Cherpitel, 2000b [18]	Santa Clara, USA	Prospective cohort	ED patients ≥ 18 years (*n* = 1952)	RAPS4 (1)	Not reported	Alcohol	ICD-10 and DSM-IV current (last 12 months) alcohol dependence (CIDI)	0.93	0.87
				RAPS4 (1)			ICD-10 and DSM-IV current (last 12 months) harmful drinking/alcohol abuse	0.55	0.79
Cherpitel, 2000c [20]	Pachuca, Mexico and Santa Clara, USA	Prospective cohort	ED patients ≥ 18 years (*n* = 1624 [Pachuca]; 733 [Santa Clara])		*Study reports on same population, screening tools (CAGE, BMAST, AUDIT, TWEAK, RAPS, TRAUMA, REPORT, 5 MONTHLY, HOLD), and reference standard as Cherpitel, 2000a*				
Cherpitel, 2001a [21]	Pachuca, Mexico and Santa Clara, USA	Prospective cohort	Hispanic ED patients ≥ 18 years (up to 65 years in Pachuca, Mexico) (*n* = 1511 [Pachuca]; 586 [Santa Clara]; not all included in analyses)		*Study reports on same population, screening tools (CAGE, TWEAK, HOLD), and reference standard as Cherpitel, 2000a*				
Cherpitel, 2001b [17]	Jackson & Hinds County, USA	Prospective cohort	ED patients ≥ 18 years (*n* = 1498)	CAGE (2)	Trained interviewers	Alcohol	ICD-10 or DSM-IV current alcohol dependence (CIDI)	0.89	0.94
				BMAST (6)				0.28	0.99
				AUDIT (8)				0.93	0.94
				TWEAK (3)				0.89	0.92
				RAPS (1)				0.97	0.86
				HOLD (5)				0.83	0.82
				5 MONTHLY (1)				0.74	0.92
Cherpitel, 2003 [28]	USA	Prospective cohort	ED patients ≥ 18 years (*n* = 412)	AUDIT (8)	Research assistants	Alcohol	DSM-IV alcohol abuse (CIDI)	0.79	0.95
				RAPS4 (1)				0.82	0.93
				RAPS4-QF (1 or both QF questions positive)				0.98	0.83
				AUDIT (8)			DSM-IV alcohol dependence (CIDI)	0.89	0.92
				RAPS4 (1)				0.89	0.90
Cherpitel, 2005 [29]	Poland	Prospective cohort	ED patients ≥ 18 years (*n* = 1492)	RAPS4 (1 [male])	Trained interviewers	Alcohol	ICD-10 or DSM-IV alcohol dependence (CIDI)	0.92	0.83
				RAPS4 (1 [female])				1.00	0.97
				CAGE (2 [male])				0.66	0.94
				CAGE (2 [female])				0.90	0.99
				AUDIT (8 [male])				0.94	0.81
				AUDIT (8 [female])				1.00	0.98
				RAPS4 (1 [male])			ICD-10 or DSM-IV alcohol abuse/harmful drinking (CIDI)	0.57	0.82
				RAPS4 (1 [female])				0.42	0.97
				RAPS4-QF (1 [male])				0.92	0.43
				RAPS4-QF (1 [female])				0.84	0.86
				CAGE (2 [male])				0.28	0.92
				CAGE (2 [female])				0.21	0.96
				AUDIT (8 [male])				0.59	0.80
				AUDIT (8 [female])				0.37	0.97
				RAPS4 (1 [male])			ICD-10 or DSM-IV alcohol dependence or abuse/harmful drinking (CIDI)	0.70	0.88
				RAPS4 (1 [female])				0.59	0.98
				RAPS4-QF (1 [male])				0.94	0.46
				RAPS4-QF (1 [female])				0.9	0.87
				CAGE 2 (2 [male])				0.42	0.97
				CAGE 2 (2 [female])				0.41	0.99
				AUDIT (8 [male])				0.71	0.86
				AUDIT (8 [female])				0.56	0.97
Cremonte, 2010 [30]	Mar del Plata, Argentina, Pachuca, Mexico and Santa Clara, USA	Prospective cohort	ED patients ≥ 18 years who reported having consumed ≥ 1 drink in last 12 months (current drinkers) (*n* = 662 [Buenos Aires, Argentina]; 559 [Pachuca, Mexico]; 884 [Santa Clara, USA])	AUDIT (8 [Argentina])	Trained interviewers	Alcohol	DSM-IV alcohol dependence (CIDI)	0.93	0.8
				AUDIT (8 [Mexico])				0.92	0.98
				AUDIT (8 [USA])				0.94	0.81
				CAGE (1 [Argentina])				0.75	0.87
				CAGE (1 [Mexico])				0.92	0.64
				CAGE (1 [USA])				0.96	0.68
				RAPS4 (1 [Argentina])				0.89	0.87
				RAPS4 (1 [Mexico])				0.92	0.98
				RAPS4 (1 [USA])				0.95	0.75
				TWEAK (2 [Argentina])				0.98	0.67
				TWEAK (2 [Mexico])				0.9	0.98
				TWEAK (2 [USA])				0.91	0.81
Cremonte, 2008 [31]	Mar de Plata, Argentina	Prospective cohort	ED patients ≥ 18 years who reported having consumed ≥ 1 drink in last 12 months (current drinkers) (*n* = 779 [92% completion]; 643 current drinkers analyzed)	AUDIT (8)	Trained interviewers	Alcohol	DSM-IV alcohol abuse (CIDI)	0.65	0.77
				CAGE (1)				0.48	0.82
				BMAST (6)				0.04	0.95
				RAPS4 (1 or both quantity and frequency questions)				0.86	0.64
				TWEAK (2)				0.82	0.65
				AUDIT (8)			ICD-10 harmful drinking (CIDI)	0.68	0.77
				CAGE (1)				0.51	0.82
				BMAST (6)				0.04	0.95
				RAPS4 (1 or both quantity and frequency questions)				0.88	0.68
				TWEAK (2)				0.66	0.62
				BMAST (6)			DSM-IV or ICD-10 alcohol dependence (CIDI)	0.44	0.99
					*Study also reports on same population, certain screening tools (AUDIT, CAGE, RAPS4, TWEAK), and reference standard of alcohol dependence as Cremonte, 2010 – results not repeated)*				
Friedmann, 2001 [32]	USA	Prospective cohort	ED patients ≥ 18 years (*n* = 504 approached; 395 completed screening questionnaire [35 unresponsive or overlooked]; 250 received CIDI gold standard interview)	CAGE (1)	Research assistants	Alcohol	Alcohol abuse OR dependence (prior 12 months) (CIDI)	0.69	0.86
				CAGE (2)				0.49	0.90
				Augmented CAGE (2)				0.72	0.85
				Quantity-Frequency (> 14 drinks/week [Male]; > 7 drinks/week [Female])				0.25	0.94
				Maximum on an occasion (> 4 drinks/occasion [Male]; > 3 drinks/occasion [Female])				0.46	0.90
				Quantity-Frequency OR Maximum on an occasion (as per thresholds above)				0.48	0.89
				NIAAA Strategy (CAGE, Quantity-Frequency, or Maximum on an occasion as per thresholds above)				0.83	0.84
				CAGE (1)			Alcohol abuse OR dependence (lifetime) (CIDI)	0.69	0.83
				CAGE (2)				0.48	0.87
				Augmented CAGE (2)				0.73	0.81
				Quantity-Frequency (> 14 drinks/week [Male]; > 7 drinks/week [Female])				0.24	0.92
				Maximum on an occasion (> 4 drinks/occasion [Male]; > 3 drinks/occasion [Female])				0.41	0.87
				Quantity-Frequency OR Maximum on an occasion (as per thresholds above)				0.43	0.87
				NIAAA Strategy (CAGE, Quantity-Frequency, or Maximum on an occasion as per thresholds above)				0.81	0.8
Galicia, 2016 [33]	Spain	Retrospective cohort	ED patients presenting due to recent cocaine use from Jan 1-Dec 31, 2010 (“recent” not defined) (*n* = 933)	MARRIED-cocaine (210)	Trained reviewer	Cocaine	Outcome: ED revisit (timeframe not specified)	0.46	0.83
Geneste, 2012 [34]	France	Prospective cohort	ED patients 18–80 years presenting with acute alcohol intoxication as principal or additional diagnosis AND blood alcohol level ≥ 0.8 g/L (*n* = 164)	AUDIT (12)	Trained interviewers	Alcohol	DSM-IV alcohol abuse/harmful drinking or alcohol dependence (MINI)	0.88	0.88
				CAGE (3)				0.84	0.76
				RAPS4 (1)				0.95	0.65
				RAPS4-QF (3)				0.89	0.77
				AUDIT (18)			DSM-IV alcohol dependence (MINI)	0.80	0.83
				CAGE (3)				0.89	0.61
				RAPS4 (2)				0.64	0.84
				RAPS4-QF (3)				0.92	0.53
Giguere, 2017 [35]	Canada	Prospective cohort	Adults with psychiatric complaints in an emergency setting (*n* = 912)	DAST	Self-report on electronic tablet computer	Multiple drugs	Any ICD-10 substance use disorder (medical record review)	Men AUC = 0.794; optimal cut-off = 4 (sensitivity and specificity at this cut-off not provided) Women AUC = 0.748; optimal cut-off = 2 (sensitivity and specificity at this cut-off not provided)	
Kelly, 2004 [36]	USA	Prospective cohort	ED patients ≥ 12 and ≤ 20 years; only patients ≥ 18 years analyzed (*n* = 246; 191 consented for follow-up)	AUDIT (10)	Patient questionnaire “self-report” At follow-up: primary investigator or Master’s-level clinical assessors	Alcohol	DSM-IV alcohol abuse or dependence (SCID)	0.82	0.78
				CAGE (1)				0.66	0.58
				CAGE (2)				0.53	0.78
				CRAFFT (3)				0.82	0.67
				RAPS-QF (3)				0.82	0.55
Kelly, 2009 [37]	USA	Prospective cohort	ED patients ≥ 12 and ≤ 20 years; only patients ≥ 18 years analyzed (*n* = 419 participated in ED study; 340 [81%] consented for follow-up)	AUDIT-C (6)	Patient questionnaire	Alcohol	DSM-IV-TR alcohol use disorder (SCID)	0.74	0.77
				FAST (3)				0.83	0.73
				RAPS4-QF (3)				0.79	0.72
				RUFT-Cut (3)				0.80	0.68
				CRAFFT (3)				0.69	0.73
				DSM-IV-2 (1)				0.88	0.90
Lee, 2019 [38]	South Korea	Prospective cohort	ED patients ≥ 18 and ≤ 90 years presenting post suicide attempts, “suicide attempters” (*n* = 621)	AUDIT-C (4 [Male])	Self-report questionnaire	Alcohol	DSM-IV-TR alcohol use disorder (chart review)	0.97	0.90
				AUDIT-C (8 [Female])				0.77	0.91
Meneses-Gaya, 2010a [39]	Brazil	Prospective cohort	Patients ≥ 18 years from a Psychosocial Care Center for Alcohol and Drugs and from the ED (*n* = 530; 449 ED patients)	AUDIT (9)	Assistant psychologist with extensive training in use of rating scales	Alcohol	DSM-IV alcohol abuse (SCID)	0.88	0.87
				AUDIT-3 (2)				0.84	0.84
				AUDIT-4 (7)				0.91	0.84
				AUDIT-C (6)				0.9	0.83
				AUDIT-QF (5)				0.89	0.81
				AUDIT-PC (6)				0.84	0.89
				CAGE (1)				0.78	0.81
				FAST (2)				0.92	0.81
				Five-Shot (2)				0.93	0.79
				AUDIT (13)			DSM-IV alcohol dependence (SCID)	0.87	0.94
				AUDIT-3 (3)				0.83	0.9
				AUDIT-4 (10)				0.86	0.92
				AUDIT-C (8)				0.88	0.88
				AUDIT-QF (6)				0.86	0.87
				AUDIT-PC (8)				0.9	0.91
				CAGE (1)				0.85	0.8
				FAST (6)				0.82	0.94
				Five-Shot (3)				0.86	0.9
				AUDIT (9)			DSM-IV alcohol abuse AND dependence (SCID)	0.88	0.92
				AUDIT-3 (2)				0.85	0.89
				AUDIT-4 (7)				0.91	0.89
				AUDIT-C (7)				0.91	0.85
				AUDIT-QF (5)				0.89	0.86
				AUDIT-PC (7)				0.84	0.94
				CAGE (1)				0.78	0.85
				FAST (2)				0.92	0.85
				Five-Shot (2)				0.93	0.83
Meneses-Gaya, 2010b [40]	Brazil	Prospective cohort		*Study reports on same population, screening tools (AUDIT, FAST), and reference standard as Meneses-Gaya, 2010a*					
Neumann, 2004 [41]	Germany	Prospective cohort	Trauma patients ≥ 18 years (*n* = 1927)	AUDIT (8 [Male])	Self-administered on laptop computer	Alcohol	ICD-10 alcohol dependence, harmful alcohol use, or high-risk drinking (face-to-face diagnostic interviews)	0.75	0.84
				AUDIT (5 [Female])				0.84	0.81
Neumann, 2009 [42]	Germany	Prospective cohort	ED patients ≥ 18 years presenting with acute injury (*n* = 1233)	AUDIT (8 [Male])	Self-administered on laptop computer	Alcohol	ICD-10 alcohol dependence, harmful alcohol use, or high-risk drinking (face-to-face diagnostic interviews)	0.75	0.84
				AUDIT (5 [Female])				0.79	0.85
Sattler, 2019 [43]	USA	Prospective cohort	ED patients ≥ 18 and ≤ 91 years (*n* = 259)	Computerized dynamic screening instrument (Optimized cut-off)	Self-administered with clinical psychology graduate student researchers nearby to answer questions, clarify meaning of items upon request, or read items aloud and record participants' responses if they were incapable of doing so themselves	Multiple drugs	Drug abuse (CIDI)	0.75	0.98
				Computerized dynamic screening instrument (Optimized cut-off)		Alcohol	Alcohol abuse (CIDI)	0.50	1.00
Seale, 2018 [44]	USA	Prospective cohort	ED patients ≥ 18 years (*n* = 221)	Q1. Validated Single Drug Screening Question – How many times in the past year have you used an illegal drug or used a prescription medication for nonmedical reasons (for example, because of the experience or feeling it caused)? (Yes)	Research assistants	Illegal drugs or, non-medical use of prescription medications	Illicit drug use (MINI)	0.65	0.99
				Q2. Single Drug Screening Question (SDSQ) – In the last twelve months, did you smoke pot (marijuana), use another street drug, or use a prescription painkiller, stimulant, or sedative for a non-medical reason? (Yes)		Marijuana, street drugs, or non-medical use of prescription medications		0.68	1.00
				Q3. Single Drug Screening Question (SDSQ) – In the last twelve months, did you smoke pot (marijuana), use another street drug, or use a prescription medication ‘recreationally’ (just for the feeling, or using more than prescribed)? (Yes)		Marijuana, street drugs, or recreational use of prescription medications		0.71	0.99
				Q4. Single Drug Screening Question (SDSQ) – In the last twelve months, on how many days did you smoke pot (marijuana), use another street drug, or use a prescription medication ‘recreationally’ (just for the feeling, or more than prescribed)? (Yes)		Marijuana, street drugs, or recreational use of prescription medications		0.68	0.99
Singh, 2015 [45]	India	Prospective cohort	Adult emergency medical services patients who consumed any alcohol in the previous year (*n* = 211; 100 [Cohort 1], 111 [Cohort 2])	Hindi 29-item alcohol screening questionnaire: 5 items predictive (3)	Physicians on duty	Alcohol	ICD-10 alcohol dependence (MINI)	0.78	0.89
van der Westhuizen, 2016 [46]	South Africa	Prospective cohort	Emergency centre patients ≥ 18 years presenting with acute injury due to assault or unintentional causes (falls, burns, etc.) (*n* = 200)	ASSIST (42)	Trained interviewers	Multiple drugs	ICD-10 and DSM-IV total substance abuse and dependence (MINI)	0.64	0.61
				ASSIST (14.5)			ICD-10 and DSM-IV alcohol abuse and dependence (MINI)	0.6	0.6
				ASSIST (18)			ICD-10 and DSM-IV illicit substance abuse and dependence (MINI)	0.9	0.87
Vinson, 2007 [47]	USA	Prospective cohort	ED patients with acute injury (*n* = 2517)	Alcohol questions form Diagnostic Interview Schedule, 2 constructs predictive (recurrent drinking in situations where physically hazardous, and drinking in larger amounts or over a longer period than intended)	Research staff	Alcohol	DSM-IV alcohol use disorder (AUDA)	0.96	0.85
Williams, 2001 [48]	USA	Prospective cohort	ED patients ≥ 18 years presenting within 48 h of acute injury (*n* = 2199 during ED shifts; 358 additional “missed” admitted patients; total 2517 individual patients)	Single screening question "When was the last time you had more than 4 (women)/5 (men) drinks containing alcohol?" (3 months)	Trained interviewers	Alcohol	Past-month hazardous drinking (≥ 4 drinks in 1 day or ≥ 14 in 1 week [men]; ≥ 3 in 1 day or ≥ 7 in 1 week [women]) OR past-year DSM-IV alcohol abuse/dependence (DIS)	0.86	0.86
							Hazardous drinking (≥ 4 drinks in 1 day or ≥ 14 in 1 week [men]; ≥ 3 in 1 day or ≥ 7 in 1 week [women]) (DIS)	0.94	0.83
							DSM-IV alcohol abuse/dependence (DIS)	0.83	0.72
							Hazardous drinking (≥ 4 drinks in 1 day or ≥ 14 in 1 week [men]; ≥ 3 in 1 day or ≥ 7 in 1 week [women]) OR DSM-IV alcohol abuse/dependence (DIS)	0.97	0.70
Wilson, 2020 [49]	USA	Prospective cohort	ED patients ≥ 18 years reporting pain persisting in the same body part ≥ 6 months even if unrelated to the current ED visit, or requesting a refill for opioids regardless of pain duration (*n* = 154 enrolled after initial inclusion/exclusion criteria, and 82 who were on opioids at time of ED presentation)	COMM (9)	Self-completed survey (combining COMM and two other survey instruments)	Opioids	Objective documentation of drug-aberrant behaviors on EMR and/or medical examiner databases	0.45	0.55

^a^Brousse, 2014 reported insufficient information to allow derivation of sensitivity and specificity from reported PPV and NPV values

Most studies evaluated tools designed to screen for alcohol use problems (*n* = 26). The most commonly evaluated tools were the Alcohol Use Disorders Identification Test (AUDIT; 14 studies), Cut down/Annoyed/Guilty/Eye-opener (CAGE; 13 studies), and the Rapid Alcohol Problems Screen and its derivatives (RAPS, RAPS4, RAPS4-QF; 12 studies). A minority of studies addressed drugs/substances more generally (*n* = 5 [N.B. some overlap with alcohol studies]), opioids (*n* = 3), and cocaine (*n* = 1).

### Results of individual studies and syntheses

We summarize tool sensitivities and specificities in Table 1. Studies evaluated screening tools’ test characteristics against a range of reference standards, most commonly DSM-IV alcohol abuse, dependence, and/or use disorder (*n* = 22); some studies reported on the same study population and are only summarized once. Other studies evaluated screening tool characteristics against reference standards of illicit substance use, dependence, and/or substance use disorder (DSM-IV); opioid use disorder (DSM-V); alcohol dependence, harmful alcohol use, or high-risk drinking (International Classification of Diseases 10th revision [ICD-10]); drug abuse (Composite International Diagnostic Interview [CIDI]); alcohol abuse (CIDI); and illicit drug use (MINI). Reported sensitivities amongst all screening tools ranged from 4 to 100%, and specificities from 43 to 100%.

#### Alcohol abuse/dependence

**Fig. 2:**
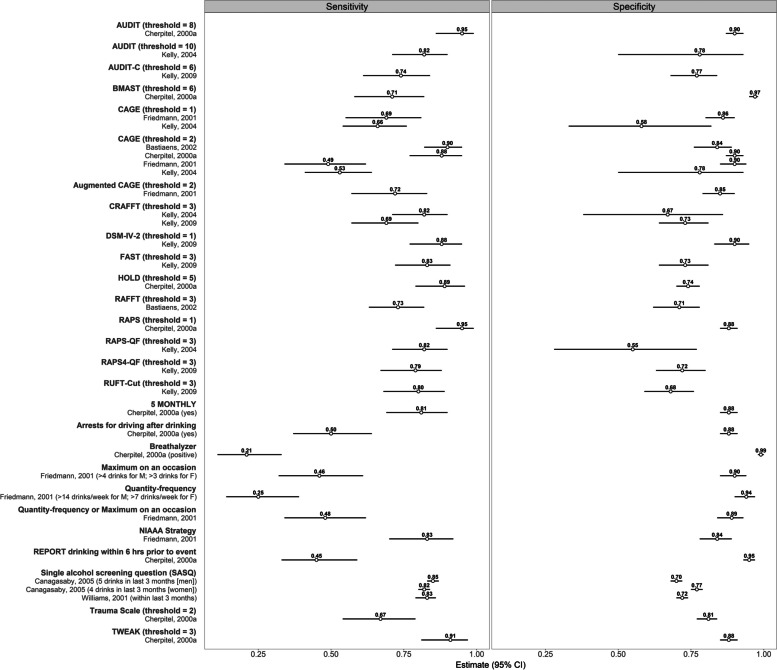
Forest plot of screening tool sensitivity and specificity for detection of DSM-IV alcohol abuse/dependence

Figure 2 displays sensitivity and specificity for six studies assessing screening tools for alcohol abuse and/or dependence in North American ED patients. Four studies assessing six tools and screening thresholds (AUDIT ≥ 8; CAGE ≥ 2; DSM-IV-2 ≥ 1; RAPS ≥ 1; National Institute on Alcohol Abuse and Alcoholism [NIAAA]; Tolerance/Worry/Eye-opener/Amnesia/K-Cut down [TWEAK] ≥ 3) reported sensitivities and specificities that were both ≥ 83% [19, 22, 32, 37]. Overall, AUDIT ≥ 8 and RAPS ≥ 1 demonstrated the highest sensitivities (95%) [18]. The tools that demonstrated the lowest sensitivities were breathalyzer (21%) [19], quantity-frequency (25%) [32], and reporting drinking within 6 h prior to event (45%) [19]. Notably, two studies evaluated a single alcohol screening question (SASQ) for problem drinking (“*When was the last time you had more than X drinks in 1 day?,” where X* = *4 for women and X* = *5 for men; within 3 months considered positive)*. The first study reported sensitivities of 85% and 82%, and specificities of 70% and 77% among men and women, respectively [26]. The second study reported a sensitivity of 83% and specificity of 72% among all screened patients [48].

#### Other substances

Five studies evaluated screening tools for general substance abuse and/or dependence (Relax/Alone/Friends/Family/Trouble [RAFFT] ≥ 3; Drug Abuse Screening Test [DAST] ≥ 4; single drug screening question; Alcohol, Smoking and Substance Involvement Screening Test [ASSIST] ≥ 42/18), and reported sensitivities ranging from 64%-90% and specificities from 61%-100% [22, 35, 43, 44, 46]. The ASSIST tool at a threshold of 18 performed best in one study, which reported sensitivity of 90% and specificity of 87% at detecting illicit substance abuse and dependence [46]. One study assessed screening for opioid misuse or use disorder using the Prescription Drug Use Questionnaire Patient Version tool (PDUQp; reported sensitivities 38%-56%, and specificities 75%-81%) [23], and two studies evaluated tools predicting aberrant behaviors related to opioid prescriptions (reported sensitivities 38%-93%, and specificities 45%-60%) [27, 49]. One study examined a screening tool for repeat ED visits among people with cocaine-related ED visits (reported sensitivity 46% and specificity 83%) [33].

We summarize components of the six alcohol screening tools that had both sensitivities and specificities ≥ 83%, the single alcohol screening question, and the ASSIST tool that performed best for general substance use and/or dependence in Additional file 2.

### Risk of bias

We summarize studies’ risk of bias in Table 2. The large majority of studies (73%, *n* = 24/33) ranked as high risk of bias on at least one domain. In the domain of patient selection, 13 studies were rated low risk of bias, seven high risk, and 13 unclear. In 19 studies, the conduct or interpretation of the screening test were high risk of bias. Most studies did not provide enough information to determine whether application of the reference standard was likely to introduce bias (*n* = 26 unclear). In the domain of patient flow (e.g., whether all patients received the reference standard, and appropriateness of interval between index test and reference standard), 15 studies were rated high risk, 15 low risk, and three unclear risk of bias.

**Table 2:** Study risk of bias

**Author, Year**	**Patient Selection**	**Index Test**	**Reference standard**	**Flow & timing**
	**Could the selection of patients have introduced bias?**	**Could the conduct or interpretation of the index test have introduced bias?**	**Could the reference standard, its conduct, or its interpretation have introduced bias?**	**Could the patient flow have introduced bias?**
Bastiaens, 2002 [22]	Unclear	Yes	Yes	Unclear
Beaudoin, 2016 [23]	Low	Yes	Unclear	Yes
Borges, 2001 [24]	Low	Unclear	Unclear	Unclear
Brousse, 2014 [25]	Unclear	Yes	Unclear	Unclear
Canagasaby, 2005 [26]	High	Unclear	Unclear	Yes
Chalmers, 2019 [27]	Unclear	Unclear	Unclear	Yes
Cherpitel, 2000a [19]	Low	No	Unclear	No
Cherpitel, 2000b [18]	Low	Yes	Unclear	No
Cherpitel, 2000c [20]	Low	No	Unclear	No
Cherpitel, 2001a [21]	Low	No	Unclear	No
Cherpitel, 2001b [17]	Unclear	No	Unclear	No
Cherpitel, 2003 [28]	Low	No	No	No
Cherpitel, 2005 [29]	Unclear	Yes	Unclear	No
Cremonte, 2010 [30]	Low	Unclear	Unclear	No
Cremonte, 2008 [31]	Low	Unclear	Unclear	Yes
Friedmann, 2001 [32]	Low	No	Unclear	No
Galicia, 2016 [33]	Low	Yes	Unclear	Yes
Geneste, 2012 [34]	Unclear	Yes	Unclear	Yes
Giguere, 2017 [35]	Unclear	Yes	Unclear	Yes
Kelly, 2004 [36]	High	Yes	Unclear	No
Kelly, 2009 [37]	High	Yes	Unclear	No
Lee, 2019 [38]	Unclear	Yes	Yes	No
Meneses-Gaya, 2010a [39]	Low	Yes	Unclear	Yes
Meneses-Gaya, 2010b [40]	Unclear	Yes	Unclear	Yes
Neumann, 2004 [41]	Low	Yes	Unclear	Yes
Neumann, 2009 [42]	High	Yes	Yes	Yes
Sattler, 2019 [43]	Unclear	Unclear	Yes	Yes
Seale, 2018 [44]	Unclear	No	Unclear	No
Singh, 2015 [45]	High	Yes	Yes	Yes
van der Westhuizen, 2016 [46]	Unclear	Yes	Unclear	Yes
Vinson, 2007 [47]	Unclear	Yes	No	No
Williams, 2001 [48]	High	Yes	Unclear	No
Wilson, 2020 [49]	High	No	Unclear	Yes

## Discussion

### Interpretation of findings

In our systematic review, six screening tools at specific thresholds concurrently demonstrated both sensitivities and specificities ≥ 83% in identifying ED patients with alcohol abuse and/or dependence. Although study heterogeneity precluded meta-analysis, our descriptive summaries show that multiple tools performed comparably. Given that no single tool appeared superior to others, the feasibility and logistics of ED screening approaches are important considerations. Simplicity, ease of recall, and clinicians’ ability to apply tools consistently and to efficiently integrate them into existing workflows are paramount (e.g., single question screens). Additionally, screening tools with higher sensitivity level should be prioritized to avoid false negatives and to increase detection of people at risk from harmful substance use who could benefit from interventions initiated or arranged from EDs. Based on our review, ED clinicians should prioritize the six tools that concurrently demonstrated both sensitivities and specificities ≥ 83% in ED settings, particularly those with the highest reported sensitivities of 95% (AUDIT ≥ 8 and RAPS ≥ 1). Screening tools that reported the lowest sensitivities (breathalyzer, quantity-frequency, and reporting drinking within 6 h prior to event) should be discontinued due to unacceptable risk of false negatives. Due to simplicity and efficiency, we recommend that ED clinicians consider integrating the single alcohol screening question (SASQ) for problem alcohol use into ED patients’ assessments (*When was the last time you had more than X drinks in 1 day?,” where X* = *4 for women and X* = *5 for men; within 3 months considered positive*). SASQ demonstrated sensitivities of 82–85% and specificities of 70–77% in two studies.

Five studies examined screening tools for general substance use. Although variable, reported sensitivities up to 90% and specificities up to 100% indicate that tools can accurately rule out and rule in harmful substance use in EDs. One tool (ASSIST ≥ 18) demonstrated sensitivity of 90% and specificity of 87% at detecting illicit substance use and dependence and could be considered in the ED setting on the basis of this data. However, a lack of studies evaluating screening tools for general substance use with common reference standards preclude recommendations. Furthermore, all results must be interpreted with caution given that included studies had predominantly high or uncertain risk of bias in key domains related to diagnostic studies. Low numbers of studies examining other specific substances preclude further interpretations.

ED providers face multiple competing demands, staffing shortages [50], resource strains [51], and burnout [52]. Screening must therefore seamlessly pair identification of high-risk patients with improved access to addictions resources that support frontline providers’ ability to provide improved patient care, rather than increase their workload.

### Comparison to previous studies

Our results corroborate findings from a previous review supporting the utility of ED screening for substance use disorders, particularly for alcohol [11]. Our findings provide renewed support for recommendations from the American College of Emergency Physicians and Canadian Association of Emergency Physicians emphasizing importance of ED substance use screening and treatment initiation [12, 13]. Our results should be interpreted in light of evidence supporting the effectiveness of brief substance use interventions in EDs [53], as screening is a crucial step in initiating a spectrum of supports for high-risk individuals once identified.

### Strengths and limitations

Our extraction of sensitivity and specificity data from included studies, either reported directly by authors or that we have derived from the presented data, is a strength. By collating data on comparative performance, we hope to inform clinicians’ and policy-makers’ decisions regarding which tools may be applicable to their local settings. Our systematic methodology, adherence to PRISMA guidelines, and use of the QUADAS-2 quality assessment tool specific to diagnostic accuracy studies strengthen our study’s rigour. Our review is limited by included studies’ heterogeneity, which prevented meta-analysis. Studies covered very different populations from various countries, and evaluated different subsets of ED patients (e.g., general versus specific presentations [psychiatric, intoxication, injury]), which limit generalizability. Moreover, only two studies evaluated screening by ED staff (most evaluated screening by trained research personnel), limiting real-world generalizability. Moreover, the majority of studies were ranked as uncertain or high risk of bias in one or more of the specific QUADAS-2 domains. Clinicians should consider studies’ risk of bias assessments globally in judging the applicability of reported screening tools to their own setting. For example, EDs seeking to implement strategies reported in studies that have high risk of bias in the domain of conduct or interpretation of the index test should carefully consider how their implementation of the reported tools (e.g., personnel applying the screens, and screening procedures/settings) compare to the original studies’ approaches. Furthermore, a paucity of studies evaluating substances other than alcohol limit conclusions. Finally, the impact of screening in the ED on patient substance use is not known. Nonetheless, our results offer a crucial update on ED approaches to screening, which is particularly needed given rising substance-related ED presentations in North America [2].

### Clinical implications

Our results demonstrate that screening can accurately identify people with harmful alcohol and substance use in EDs. Interpreted in the context that one in 11 ED visits are made by people with substance use disorders [3], ED screening may capitalize on crucial opportunities to identify high-risk individuals who may not present elsewhere to the healthcare system. In addition to brief intervention and referral [54], emerging evidence supports the feasibility and effectiveness of ED treatment initiation (e.g., naltrexone for alcohol use disorder, buprenorphine/naloxone for opioid use disorder, linkage to urgent follow-up and community services [55–57]. The use of accurate screening tools will likely increase the number of patients who are initiated on such treatments in the ED.

### Research implications

Future research should address gaps we have identified, particularly evaluation of screening tools for substances other than alcohol (e.g., opioids, stimulants). Additionally, in most studies screening was applied by trained research staff: research must validate whether tools perform well when applied by frontline practitioners, and how they can be best integrated into actual ED workflows from an implementation and quality improvement perspective. Data on patient outcomes following ED screening and initiation or referral to treatment are also lacking.

## Conclusions

Six screening tools at various thresholds concurrently demonstrated both sensitivities and specificities ≥ 83% at detecting alcohol abuse and/or dependence when applied in EDs. Based on our review, tools with the highest sensitivities (AUDIT ≥ 8 and RAPS ≥ 1) and that prioritize simplicity and efficiency (single screening question for problem alcohol use [SASQ]) should be prioritized, while those with unacceptably low sensitivities (breathalyzer, quantity-frequency, and reporting drinking within 6 h prior to event) should be discontinued due to risk of false negatives. Practitioners and policy-makers should consider integrating substance use screening into workflows that combine identification of high-risk patients with improved access to addictions resources and enhanced supports for frontline clinicians.

### Supplementary Information

**Supplementary Material 1. **

**Supplementary Material 2. **

**Supplementary Material 3. **

## Data Availability

Template data collection forms and quality assessment forms are available in Additional file 3. Data extracted from included studies and data used for all analyses are available from authors upon request.

## Abbreviations

ASSIST: Alcohol, Smoking and Substance Involvement Screening Test
AUDIT: Alcohol Use Disorders Identification Test
CAGE: Cut down/Annoyed/Guilty/Eye-opener
CINAHL: Cumulative Index to Nursing and Allied Health Literature
DAST: Drug Abuse Screening Test
DSMIV: Diagnostic and Statistical Manual of Mental Disorders, 4th Edition
ED: Emergency department
HaPI: Health and Psychosocial Instruments
MEDLINE: Medical Literature Analysis and Retrieval System Online
MeSH: Medical Subject Headings
NIAAA: National Institute on Alcohol Abuse and Alcoholism
PDUQp: Prescription Drug Use Questionnaire Patient Version
PRISMA: Preferred Reporting Items for Systematic Reviews and Meta-Analyses
QUADAS-2: Quality Assessment of Diagnostic Accuracy Studies-2
RAFFT: Relax/Alone/Friends/Family/Trouble
RAPS: Rapid Alcohol Problems Screen
TWEAK: Tolerance/Worry/Eye-opener/Amnesia/K-Cut down

## References

[1] Urbanoski K, Cheng J, Rehm J, Kurdyak P (2018). Frequent use of emergency departments for mental and substance use disorders. Emerg Med J.

[2] Lavergne MR, Shirmaleki M, Loyal JP, Jones W, Nicholls TL, Schutz CG (2022). Emergency department use for mental and substance use disorders: descriptive analysis of population-based, linked administrative data in British Columbia, Canada. BMJ Open.

[3] Suen LW, Makam AN, Snyder HR, Repplinger D, Kushel MB, Martin M, et al. National prevalence of alcohol and other substance use disorders among emergency department visits and hospitalizations: NHAMCS 2014–2018. J Gen Intern Med. 2022:37(10):2420–8. 10.1007/s11606-021-07069-w PMC8436853 34518978

[4] White AM, Slater ME, Ng G, Hingson R, Breslow R (2018). Trends in alcohol-related emergency department visits in the United States: results from the Nationwide Emergency Department Sample, 2006 to 2014. Alcohol Clin Exp Res.

[5] Peterson C, Li M, Xu L, Mikosz CA, Luo F (2021). Assessment of annual cost of substance use disorder in US hospitals. JAMA Netw Open.

[6] Canadian Substance Use Costs and Harms Working Group (2023). Canadian substance use costs and harms 2007–2020. Prepared by the Canadian Institute for Substance Use Research and the Canadian Centre on Substance Use and Addiction.

[7] Myran DT, Hsu AT, Smith G, Tanuseputro P (2019). Rates of emergency department visits attributable to alcohol use in Ontario from 2003 to 2016: a retrospective population-level study. CMAJ.

[8] Moe J, Camargo CA, Jelinski S, Erdelyi S, Brubacher J, Rowe BH (2018). Epidemiologic trends in substance and opioid misuse-related emergency department visits in Alberta: a cross-sectional time-series analysis. Can J Public Health.

[9] Canadian Centre for Substance Use and Addiction (2019). Changes in stimulant use and related harms: Focus on Methamphetamine and Cocaine (CCENDU Bulletin).

[10] Otterstatter MC, Crabtree A, Dobrer S, Kinniburgh B, Klar S, Leamon A, et al. Patterns of health care utilization among people who overdosed from illegal drugs: a descriptive analysis using the BC Provincial Overdose Cohort. Health Promot Chronic Dis Prev Can. 2018;38(9):328–33. 10.24095/hpcdp.38.9.04 PMC6169704 30226726

[11] Hawk K, D’Onofrio G (2018). Emergency department screening and interventions for substance use disorders. Addict Sci Clin Pract.

[12] American College of Emergency Physicians (2005). Alcohol screening in the emergency department. Ann Emerg Med.

[13] Koh JJ, Klaiman M, Miles I, Cook J, Kumar T, Sheikh H (2020). CAEP position statement: emergency department management of people with opioid use disorder. CJEM.

[14] Page MJ, McKenzie JE, Bossuyt PM, Boutron I, Hoffmann TC, Mulrow CD (2021). The PRISMA 2020 statement: an updated guideline for reporting systematic reviews. BMJ.

[15] Covidence systematic review software. Veritas Health Innovation, Melbourne, Australia. Cited 2021-2023. Available at: www.covidence.org.

[16] Whiting PF, Rutjes AW, Westwood ME, Mallett S, Deeks JJ, Reitsma JB (2011). QUADAS-2: a revised tool for the quality assessment of diagnostic accuracy studies. Ann Intern Med.

[17] Cherpitel C. Screening for alcohol problems: a comparison of instrument performance among black emergency department and primary care patients. J Subst Use. 2001b;5(4):290–7.

[18] Cherpitel CJ. A brief screening instrument for problem drinking in the emergency room: the RAPS4. Rapid alcohol problems screen. J Stud Alcohol. 2000b;61(3):447–9. 10.15288/jsa.2000.61.447 10807217

[19] Cherpitel CJ, Borges G. Performance of screening instruments for alcohol problems in the ER: a comparison of Mexican-Americans and Mexicans in Mexico. Am J Drug Alcohol Abuse. 2000a;26(4):683–702. 10.1081/ada-100101902 11097199

[20] Cherpitel CJ, Borges G. Screening instruments for alcohol problems: a comparison of cut points between Mexican American and Mexican patients in the emergency room. Subst Use Misuse. 2000c;35(10):1419–30. 10.3109/10826080009148223 10921432

[21] Cherpitel CJ, Borges G, Medina-Mora ME. Screening for alcohol problems: A comparison of instrument performance between the ER and the general population among Mexican Americans in the US and Mexicans in Mexico. Addict Res Theory. 2001a;9(1):59–72.

[22] Bastiaens L, Riccardi K, Sakhrani D (2002). The RAFFT as a screening tool for adult substance use disorders. Am J Drug Alcohol Abuse.

[23] Beaudoin FL, Merchant RC, Clark MA (2016). Prevalence and detection of prescription opioid misuse and prescription opioid use disorder among emergency department patients 50 years of age and older: performance of the prescription drug use questionnaire, patient version. Am J Geriatr Psychiatry.

[24] Borges G, Cherpitel CJ. Selection of screening items for alcohol abuse and alcohol dependence among Mexicans and Mexican Americans in the emergency department. J Stud Alcohol. 2001;62(3):277–85. 10.15288/jsa.2001.62.277 11414336

[25] Brousse G, Arnaud B, Geneste J, Pereira B, De Chazeron I, Teissedre F, et al. How CAGE, RAPS4-QF, and AUDIT Can Help Practitioners for Patients Admitted with Acute Alcohol Intoxication in Emergency Departments? Front Psychiatry. 2014;5:72. 10.3389/fpsyt.2014.00072 PMC4067695 25009509

[26] Canagasaby A, Vinson DC (2005). Screening for hazardous or harmful drinking using one or two quantity–frequency questions. Alcohol Alcohol.

[27] Chalmers CE, Mullinax S, Brennan J, Vilke GM, Oliveto AH, Wilson MP (2019). Screening tools validated in the outpatient pain management setting poorly predict opioid misuse in the emergency department: a pilot study. J Emerg Med.

[28] Cherpitel CJ, Bazargan S. Screening for alcohol problems: comparison of the audit, RAPS4 and RAPS4-QF among African American and Hispanic patients in an inner city emergency department. Drug Alcohol Depend. 2003;71(3):275–80. 10.1016/s0376-8716(03)00140-6 12957345

[29] Cherpitel CJ, Ye Y, Moskalewicz J, Swiatkiewicz G. Screening for alcohol problems in two emergency service samples in Poland: comparison of the RAPS4, CAGE and AUDIT. Drug Alcohol Depend. 2005;80(2):201–7. 10.1016/j.drugalcdep.2005.03.025 15896929

[30] Cremonte M, Ledesma RD, Cherpitel CJ, Borges G. Psychometric properties of alcohol screening tests in the emergency department in Argentina, Mexico and the United States. Addict Behav. 2010;35(9):818–25. 10.1016/j.addbeh.2010.03.021 PMC2920125 20472341

[31] Cremonte M, Cherpitel CJ. Performance of screening instruments for alcohol use disorders in emergency department patients in Argentina. Subst Use Misuse. 2008;43(1):125–38. 10.1080/10826080701212337 18189209

[32] Friedmann PD, Saitz R, Gogineni A, Zhang JX, Stein MD (2001). Validation of the screening strategy in the NIAAA" Physicians' guide to helping patients with alcohol problems". J Stud Alcohol.

[33] Galicia M, Nogué S, Miró Ò (2016). MARRIED-cocaine score: validating a tool for detecting the risk of ED revisit in cocaine users. Emerg Med J.

[34] Geneste J, Pereira B, Arnaud B, Christol N, Liotier J, Blanc O, et al. CAGE, RAPS4, RAPS4-QF and AUDIT screening tests for men and women admitted for acute alcohol intoxication to an emergency department: are standard thresholds appropriate? Alcohol and Alcoholism (Oxford, Oxfordshire). 2012;47(3):273–81. 10.1093/alcalc/ags027 PMC3331621 22414922

[35] Giguère C-É, Potvin S, Consortium S (2017). The Drug Abuse Screening Test preserves its excellent psychometric properties in psychiatric patients evaluated in an emergency setting. Addict Behav.

[36] Kelly TM, Donovan JE, Chung T, Cook RL, Delbridge TR. Alcohol use disorders among emergency department-treated older adolescents: a new brief screen (RUFT-Cut) using the AUDIT, CAGE, CRAFFT, and RAPS-QF. Alcohol Clin Exp Res. 2004;28(5):746–53. 10.1097/01.alc.0000125346.37075.85 15166649

[37] Kelly TM, Donovan JE, Chung T, Bukstein OG, Cornelius JR (2009). Brief screens for detecting alcohol use disorder among 18–20 year old young adults in emergency departments: Comparing AUDIT-C, CRAFFT, RAPS4-QF, FAST, RUFT-Cut, and DSM-IV 2-Item Scale. Addict Behav.

[38] Lee J, Min S, Ahn JS, Kim H, Cha YS, Oh E, et al. Identifying alcohol problems among suicide attempters visiting the emergency department. BMC Psychiatry. 2019;19(1):350. 10.1186/s12888-019-2347-5 PMC6842213 31703656

[39] Meneses-Gaya C, Zuardi AW, Loureiro SR, Hallak JEC, Trzesniak C, de Azevedo Marques JM, et al. Is the full version of the AUDIT really necessary? Study of the validity and internal construct of its abbreviated versions. Alcohol Clin Exp Res. 2010a;34(8):1417–24. 10.1111/j.1530-0277.2010.01225.x 20491736

[40] Meneses-Gaya C, Crippa JAS, Zuardi AW, Loureiro SR, Hallak JEC, Trzesniak C, et al. The fast alcohol screening test (FAST) is as good as the AUDIT to screen alcohol use disorders. Subst Use Misuse. 2010b;45(10):1542–57. 10.3109/10826081003682206 20590374

[41] Neumann T, Neuner B, Gentilello LM, Weiss-Gerlach E, Mentz H, Rettig JS, et al. Gender differences in the performance of a computerized version of the alcohol use disorders identification test in subcritically injured patients who are admitted to the emergency department. Alcohol Clin Exp Res. 2004;28(11):1693–701. 10.1097/01.alc.0000145696.58084.08 15547456

[42] Neumann T, Gentilello LM, Neuner B, Weiss-Gerlach E, Schürmann H, Schröder T, et al. Screening trauma patients with the alcohol use disorders identification test and biomarkers of alcohol use. Alcohol Clin Exp Res. 2009;33(6):970–6. 10.1111/j.1530-0277.2009.00917.x 19302090

[43] Sattler AF, Bentley JP, Young J. Validation of a brief screening instrument for adult psychopathology. Prof Psychol Res Pract. 2019;50:315–22.

[44] Seale JP, Johnson JA, Cline N, Buchanan C, Kiker C, Cochran L (2018). Drug screening and changing marijuana policy: Validation of new single question drug screening tools. Drug Alcohol Depend.

[45] Singh SM, Bhalla A, Giri OP, Sarkar S. Development of Screening Questionnaire for Detection of Alcohol Dependence. J Clin Diagn Res JCDR. 2015;9(9):VC07–10. 10.7860/JCDR/2015/11974.6438 PMC4606318 26500989

[46] Van Der Westhuizen C, Wyatt G, Williams JK, Stein DJ, Sorsdahl K (2016). Validation of the alcohol, smoking and substance involvement screening test in a low-and middle-income country cross-sectional emergency centre study. Drug Alcohol Rev.

[47] Vinson DC, Kruse RL, Seale JP. Simplifying Alcohol Assessment: Two Questions to Identify Alcohol Use Disorders. Alcohol Clin Exp Res. 2007;31(8):1392–8. 10.1111/j.1530-0277.2007.00440.x 17559544

[48] Williams R, Vinson DC. Validation of a single screening question for problem drinking. J Fam Pract. 2001;50(4):307–12. 11300981

[49] Wilson MP, Cucciare MA, Porter A, Chalmers CE, Mullinax S, Mancino M (2020). The utility of a statewide prescription drug-monitoring database vs the Current Opioid Misuse Measure for identifying drug-aberrant behaviors in emergency department patients already on opioids. Am J Emerg Med.

[50] Chervoni-Knapp T (2022). The staffing shortage pandemic. J Radiol Nurs.

[51] Kelen GD, Wolfe R, D’Onofrio G, Mills AM, Diercks D, Stern SA, et al. Emergency department crowding: the canary in the health care system. NEJM Catal Innov Care Deliv. 2021;2(5). Available at: https://catalyst.nejm.org/doi/full/10.1056/CAT.21.0217.

[52] Duong D, Vogel L (2023). Overworked health workers are “past the point of exhaustion”. Can Med Assoc J.

[53] European Monitoring Centre for Drugs and Drug Addiction. Emergency department-based brief interventions for individuals with substance- related problems: a review of effectiveness. EMCDDA Papers: Publications Office of the European Union, Luxembourg; 2016.

[54] Barata IA, Shandro JR, Montgomery M, Polansky R, Sachs CJ, Duber HC (2017). Effectiveness of SBIRT for alcohol use disorders in the emergency department: a systematic review. West J Emerg Med.

[55] Murphy CE 4th, Coralic Z, Wang RC, Montoy JCC, Ramirez B, Raven MC. Extended-release naltrexone and case management for treatment of alcohol use disorder in the emergency department. Ann Emerg Med. 2023;81(4):440–9. 10.1016/j.annemergmed.2022.08.453 36328851

[56] D'Onofrio G, O'Connor PG, Pantalon MV, Chawarski MC, Busch SH, Owens PH (2015). Emergency department-initiated buprenorphine/naloxone treatment for opioid dependence: a randomized clinical trial. JAMA.

[57] Thomas CP, Stewart MT, Tschampl C, Sennaar K, Schwartz D, Dey J (2022). Emergency department interventions for opioid use disorder: A synthesis of emerging models. J Subst Abuse Treat.

