# Supplementary material for: Screening for harmful substance use in emergency departments: a systematic review
Source: Int J Emerg Med. 2024 Apr 8;17:52. doi: 10.1186/s12245-024-00616-2 (PMC11000386; doi:10.1186/s12245-024-00616-2)
Supplement: Supplementary file 1 — Supplementary Material 1. [file 12245_2024_616_MOESM1_ESM.docx]

**Additional file 1: Search Strategy for MEDLINE (Ovid)**

**Search was executed on:** Jan. 5, 2021

1 emergency department?.ti,ab,kf. (98085)

2 emergency room?.ti,ab,kf. (20029)

3 emergency medicine.ti,ab,kf. (17428)

4 (trauma adj2 (centre or center or room? or department?)).ti,ab,kf. (15050)

5 or/1-4 (140561)

6 *Emergency Medical Services/ (30841)

7 *emergency service, hospital/ or *trauma centers/ (47219)

8 *Emergency Services, Psychiatric/ (1877)

9 *emergency medicine/ (10436)

10 or/6-9 (86813)

11 or/5,10 [ED] (186349)

12 Mass Screening/ (105533)

13 (screen or screening).ti,ab,kf. (650201)

14 brief intervention.ti,ab,kf. (3165)

15 *Early Medical Intervention/mt [Methods] (556)

16 *Counseling/mt [Methods] (3428)

17 *risk assessment/ (30846)

18 identifying patient?.ti,ab,kf. (11478)

19 SBIRT.ti,ab,kf. (440)

20 Substance Abuse Detection/mt [Methods] (5507)

21 *Health Education/mt [Methods] (8275)

22 or/12-21 [Screening] (736207)

23 11 and 22 [ED & Screening] (8429)

24 *substance-related disorders/ (70378)

25 *alcohol-related disorders/ or *alcohol-induced disorders/ or *alcohol-induced disorders, nervous system/ or *alcoholic intoxication/ or *alcoholism/ or *binge drinking/ (68775)

26 *amphetamine-related disorders/ or *cocaine-related disorders/ or *drug overdose/ or *inhalant abuse/ or *marijuana abuse/ or *narcotic-related disorders/ or *opioid-related disorders/ or *heroin dependence/ or *morphine dependence/ or *opium dependence/ or *phencyclidine abuse/ (38000)

27 *"tobacco use disorder"/ or *smoking/ or *cocaine smoking/ or *marijuana smoking/ or *tobacco smoking/ or *cigar smoking/ or *cigarette smoking/ or *vaping/ (81508)

28 *illicit drugs/ (8133)

29 *Smoking Cessation/ (20635)

30 prescription drug misuse/ (1906)

31 prescription drug overuse/ (331)

32 (substance? adj2 abuse).ti,ab,kf. (26946)

33 opioid dependence.ti,ab,kf. (2905)

34 or/24-33 (277753)

35 di.fs. [Diagnosis] (2609506)

36 th.fs. [Therapy] (1899888)

37 pc.fs. [Prevention & control] (1312212)

38 or/35-37 (5192951)

39 34 and 38 (79406)

40 11 and 22 and 39 (661)

41 limit 40 to ("adult (19 to 44 years)" or "young adult and adult (19-24 and 19-44)" or "middle age (45 to 64 years)" or "middle aged (45 plus years)" or "all aged (65 and over)" or "aged (80 and over)") (443)

42 limit 41 to yr="2000 -Current" (394)

43 limit 42 to English language (386)

44 comment/ or editorial/ or letter/ or news/ (2107186)

45 43 not 44 (383)

46 case reports.pt. (2146092)

47 45 not 46 [Remaining] (354)
