# Supplementary material for: Screening for harmful substance use in emergency departments: a systematic review
Source: Int J Emerg Med. 2024 Apr 8;17:52. doi: 10.1186/s12245-024-00616-2 (PMC11000386; doi:10.1186/s12245-024-00616-2)
Supplement: Supplementary file 2 — Supplementary Material 2. [file 12245_2024_616_MOESM2_ESM.docx]

**Additional file 2: Recommended Screening Tools**

| **Screening Tools** | **Components** | **Scoring** |
| --- | --- | --- |
| ***Alcohol*** | | |
| Alcohol Use Disorders Identification Test (AUDIT)  & AUDIT-C (short form) | 1. How often do you have a drink containing alcohol? 2. How many drinks containing alcohol do you have on a typical day when you are drinking? 3. How often do you have six or more drinks on one occasion?   Skip to Questions 9 and 10 if Total Score for Questions 2 and 3 = 0   1. How often during the last year have you found that you were not able to stop drinking once you had started? 2. How often during the last year have you failed to do what was normally expected from you because of drinking? 3. How often during the last year have you needed a first drink in the morning to get yourself going after a heavy drinking session? 4. How often during the last year have you had a feeling of guilt or remorse after drinking? 5. How often during the last year have you been unable to remember what happened the night before because you had been drinking? 6. Have you or someone else been injured as a result of your drinking? 7. Has a relative or friend or a doctor or another health worker been concerned about your drinking or suggested you cut down?   *N.B. AUDIT-C uses only the consumption items (Questions 1-3)* | Uses a 0 – 4 point Likert Scale for each question and has a range of 0-40 points. Various thresholds reported. ≥8 commonly considered a a positive screen for an alcohol use disorder, abuse and/or dependence.  AUDIT-C has a range of 0-12 points. Various thresholds (≥4, 6, 7, or 8) reported as a positive screen for alcohol use disorder, abuse, and/or dependence. |
| Cut down/Annoyed/ Guilty/Eye-opener (CAGE) | 1. Have you ever felt you should **cut down** on your drinking? 2. Have people **annoyed** you about your drinking? 3. Have you ever felt bad or **guilty** about your drinking? 4. Have you ever had a drink first thing in the morning to steady your nerves or to get rid of a hangover (**eye opener**)? | Various thresholds reported (≥1, 2, or 3). ≥2positive answers commonly considered a positive screen for alcohol abuse and/or dependence. |
| Rapid Alcohol Problems Screen (RAPS/RAPS4) | 1. During the last year, have you had a feeling of guilt or remorse after drinking? (**Remorse)** 2. During the last year, has a friend or family member ever told you about things you said or did while you were drinking that you could not remember? (**Amnesia)** 3. During the last year, have you failed to do what was normally expected from you because of drinking? **(Perform)** 4. Do you sometimes take a drink in the morning when you first get up? (**Starter**) | A positive response to ≥1of the four questions considered a positive screen for alcohol abuse and/or dependence. |
| RAPS4-QF | 1. During the last year, have you had a feeling of guilt or remorse after drinking? (**Remorse)** 2. During the last year, has a friend or family member ever told you about things you said or did while you were drinking that you could not remember? (**Amnesia)** 3. During the last year, have you failed to do what was normally expected from you because of drinking? **(Perform)** 4. Do you sometimes take a drink in the morning when you first get up? (**Starter**) 5. During the last year, have you had five or more drinks on at least one occasion? (**Quantity**) 6. During the last year, do you drink as often as once a month? (**Frequency**) | Various thresholds reported. (≥1 [+/- both QF questions positive], 2, or 3) A positive response to ≥1of the four questions commonly considered a positive screen for alcohol abuse and/or dependence. |
| Single alcohol screening question (SASQ) for problem drinking | “When was the last time you had more than X drinks in 1 day?,” where X=4 for women and X=5 for men. | Within 3 months considered positive for alcohol abuse and/or dependence. |
| DSM-IV-2-Item Scale (two items form the DSM-IV definition of alcohol abuse or dependence) | 1. “Recurrent drinking in hazardous situations” 2. “Drinking more than intended, or over a longer period than intended” | Present if patient reported in a diagnostic interview. Has a range of 0-2.  A positive response to ≥1 questions considered a positive screen for alcohol abuse and/or dependence. |
| National Institute on Alcohol Abuse and Alcoholism [NIAAA] Strategy | - CAGE questionnaire:  1. Have you ever felt you should **cut down** on your drinking? 2. Have people **annoyed** you about your drinking? 3. Have you ever felt bad or **guilty** about your drinking? 4. Have you ever had a drink first thing in the morning to steady your nerves or to get rid of a hangover (**eye opener**)?  - Quantity and frequency of drinking - Maximum consumption on an occasion. | NIAA-recommended screen positive for alcohol abuse and/or dependence if reported alcohol consumption in excess of the quantity, frequency, or maximum consumption cutpoints in the NIAAA “Physician’s Guide”:   - >14 drinks/week or maximum >4 drinks per - occasion for men - >7 drinks/week or maximum >3 drinks per occasion for women   Or if:   - CAGE ≥1 |
| Tolerance/Worry/Eye-opener/Amnesia/K-Cut down [TWEAK]≥3 | 1. **(Tolerance)** How many drinks does it take to make you feel high? (positive if ≥3 drinks) 2. Have your friends or relatives **Worried** or complained about your drinking in the past year? 3. Do you sometimes take a drink in the morning when you first get up? (**Eye-opener**) 4. Are there times when you drink and afterwards you can’t remember what you said or did? (**Amnesia**) 5. Do you sometimes feel the need to **Cut** down on your drinking? (**K or C**) | **Tolerance** and **Worried** questions score 2 points each, others score 1 point each. Score has a range of 0-7.  Various thresholds reported. ≥2 or 3 commonly considered a positive screen for alcohol dependence and/or abuse. |
| ***General Substance Use*** | | |
| ASSIST | 1. In your life, which of the following substances have you ever used (non-medical use only)? 2. In the past three months, how often have you used the substances you mentioned (first drug, second drug, etc)? 3. During the past three months, how often have you had a strong desire or urge to use (first drug, second drug, etc)? 4. During the past three months, how often has your use of (first drug, second drug, etc) led to health, social, legal or financial problems? 5. During the past three months, how often have you failed to do what was normally expected of you because of your use of (first drug, second drug, etc)? 6. Has a friend or relative or anyone else ever expressed concern about your use of (first drug, second drug, etc)? 7. Have you ever tried to cut down on using (first drug, second drug, etc) but failed? 8. Have you ever used any drug by injection (non-medical use only)?   **RESPONSE CARD \| Substances**   1. Tobacco products (cigarettes, chewing tobacco, cigars, etc.) 2. Alcoholic beverages (beer, wine, spirits, etc.) 3. Cannabis (marijuana, pot, grass, hash, etc.) 4. Cocaine (coke, crack, etc.) 5. Amphetamine-type stimulants (speed, meth, ecstasy, etc.) 6. Inhalants (nitrous, glue, petrol, paint thinner, etc.) 7. Sedatives or sleeping pills (diazepam, alprazolam, flunitrazepam, midazolam, etc.) 8. Hallucinogens (LSD, acid, mushrooms, trips, ketamine, etc.) 9. Opioids (heroin, morphine, methadone, buprenorphine, codeine, etc.) 10. Other – specify:   **RESPONSE CARD \| Frequency responses**  **Last 3 months (ASSIST questions 2 to 5)**   1. Never: not used in the last 3 months. 2. Once or twice: 1 to 2 times in the last 3 months. 3. Monthly: average of 1 to 3 times per month over the last 3 months. 4. Weekly: 1 to 4 times per week. 5. Daily or almost daily: 5 to 7 days per week.   **Response card Lifetime (ASSIST questions 6 to 8)**   1. No, never. 2. Yes, but not in the past 3 months. 3. Yes, in the past 3 months. | **Substance-specific risk scores:**   - tobacco (range 0 – 31) - alcohol (range 0 – 39) - cannabis (range 0 – 39) - cocaine (range 0 – 39) - amphetamine-type stimulants (range 0 – 39) - inhalants (range 0 – 39) - sedatives or sleeping pills (range 0 – 39) - hallucinogens (range 0 – 39) - opioids (range 0 – 39) - ‘other’ drugs (range 0 – 39).   **Alcohol:**   - Lower risk 0-10 - Moderate risk 11-26 - High risk ≥27   **All other substances:**   - Lower risk 0-3 - Moderate risk 4-26 - High risk ≥27   Various thresholds tested reported as positive screen for illicit substance abuse and/or dependence. |
