# Supplementary material for: Screening for harmful substance use in emergency departments: a systematic review
Source: Int J Emerg Med. 2024 Apr 8;17:52. doi: 10.1186/s12245-024-00616-2 (PMC11000386; doi:10.1186/s12245-024-00616-2)
Supplement: Supplementary file 3 — Supplementary Material 3. [file 12245_2024_616_MOESM3_ESM.docx]

**Additional file 3: Study Forms**

***Form 1: Study Eligibility Assessment***

| **ID** | **Study** | **Title** | **Authors** | **Published Year** | **Journal** | **Volume** | **Issue** | **Pages** | **DOI** | **REVIEWER** | **Inclusion/exclusion decision** | | **Reasons for Exclusion** | | | | **Notes** |
| --- | --- | --- | --- | --- | --- | --- | --- | --- | --- | --- | --- | --- | --- | --- | --- | --- | --- |
|  |  |  |  |  |  |  |  |  |  |  | **INCLUDE (0,1)** | **EXCLUDE (0,1)** | **P** | **I** | **O** | **D** |  |
|  |  |  |  |  |  |  |  |  |  |  |  |  |  |  |  |  |  |

***Form 2: Data Extraction***

***Study Characteristics***

| **StudyID** | **Primary author** | **Pub Year** | **Reviewer** | **Study Design (RCT, non RCT, non comparator trial, cohort)** | **Study Location (City, Country)** | **Study Time Period: over what time period was the study conducted (mos/yrs)** | **Was there a follow up period, and what was the timing of this follow up?** | **What was the source of obtaining data (Data Ascertainment), e.g. hospital database, national database, patient charts** |
| --- | --- | --- | --- | --- | --- | --- | --- | --- |
|  |  |  |  |  |  |  |  |  |

***Study Participants***

| **Inclusion Criteria for Study** | **ED Presenting complaint** | **# at baseline** | **# at follow-up** | **# in main analysis** | **# lost to follow-up** | **Average Age + SD** | **Sex (% male)** | **Gender** | **Ethnicity** | **Education level** | **Occupa-tional status** | **Marital status** | **Income level** | **Co-morbidities** | **Other** |
| --- | --- | --- | --- | --- | --- | --- | --- | --- | --- | --- | --- | --- | --- | --- | --- |
|  |  |  |  |  |  |  |  |  |  |  |  |  |  |  |  |

***Intervention***

| **Method of determining eligibility for screening, if applicable** | **How did they determine total population?** | **Screening part of SBIRT? (yes/no)** | **Screening tool(s)** | **Substance screened #1** | **Substance screened #2** | **Substance screened #3** | **Other substances screened** | **Person administering screening (e.g., ED physician, nurse, addictions doctor)** | **Person interpreting screening results (if different)** | **Other components of screening (if any)** | **Other interventions after ED screening (if any)** | **Follow-up after ED visit (e.g. addictions or GP followup)** |
| --- | --- | --- | --- | --- | --- | --- | --- | --- | --- | --- | --- | --- |
|  |  |  |  |  |  |  |  |  |  |  |  |  |

***Outcome***

| **Screening tool** | **Definition of screen + (e.g. score threshold)** | **Total screen +** | **Total screen -** | **Gold standard definition (1° outcome)** | **How was outcome determined?** | **Who assessed outcome?** | **1° outcome + (TP)** | **1° outcome - (FP)** | **1° outcome + (FN)** | **1° outcome - (TN)** |
| --- | --- | --- | --- | --- | --- | --- | --- | --- | --- | --- |
|  |  |  |  |  |  |  |  |  |  |  |

***Form 3: Risk of Bias Assessments (QUADAS-2)***

| **DOMAIN 1: Patient Selection** | | | | | | |
| --- | --- | --- | --- | --- | --- | --- |
|  |  |  |  | **RISK OF BIAS** |  | **APPLICABILITY** |
| **Describe methods of patient selection** | ***Was a consecutive or random sample of patients enrolled?*** | ***Was a case-control design avoided?*** | ***Did the study avoid inappropriate exclusions?*** | **Could the selection of patients have introduced bias?** | **Describe included patients (prior testing, presentation, intended use of index test and setting):** | **Are there concerns that the included patients do not match the review question?** |
|  |  |  |  |  |  |  |

| **DOMAIN 2: Index Text** | | | | |
| --- | --- | --- | --- | --- |
|  |  |  | **RISK OF BIAS** | **APPLICABILITY** |
| **Describe the index test and how it was conducted and interpreted:** | ***Were the index test results interpreted without knowledge of the results of the reference standard?*** | ***If a threshold was used, was it pre-specified?*** | **Could the conduct or interpretation of the index test have introduced bias?** | **Are there concerns that the index test, its conduct, or interpretation differ from the review question?** |
|  |  |  |  |  |

| **DOMAIN 3: Reference Standard** | | | | |
| --- | --- | --- | --- | --- |
|  |  |  | **RISK OF BIAS** | **APPLICABILITY** |
| **Describe the reference standard and how it was conducted and interpreted:** | ***Is the reference standard likely to correctly classify the target condition?*** | ***Were the reference standard results interpreted without knowledge of the results of the index test?*** | **Could the reference standard, its conduct, or its interpretation have introduced bias?** | **Are there concerns that the target condition as defined by the reference standard does not match the review question?** |
|  |  |  |  |  |

| **DOMAIN 4: Flow and Timing** | | | | | | |
| --- | --- | --- | --- | --- | --- | --- |
|  |  |  |  |  |  | **RISK OF BIAS** |
| **Describe any patients who did not receive the index test(s) and/or reference standard or who were excluded from the 2x2 table (refer to flow diagram):** | **Describe the time interval and any interventions between index test(s) and reference standard:** | ***Was there an appropriate interval between index test(s) and reference standard?*** | ***Did all patients receive a reference standard?*** | ***Did all patients receive the same reference standard?*** | ***Were all patients included in the analysis?*** | **Could the patient flow have introduced bias?** |
|  |  |  |  |  |  |  |
